# Preparation and Evaluation of Chitosan/PVA Based Hydrogel Films Loaded with Honey for Wound Healing Application

**DOI:** 10.3390/gels8020111

**Published:** 2022-02-11

**Authors:** Hitesh Chopra, Shabana Bibi, Sandeep Kumar, Muhammad Saad Khan, Pradeep Kumar, Inderbir Singh

**Affiliations:** 1 Chitkara College of Pharmacy, Chitkara University, Rajpura 140401, Punjab, India; chopraontheride@gmail.com; 2 Yunnan Herbal Laboratory, College of Ecology and Environmental Sciences, Yunnan University, Kunming 650091, China; shabana_bibi@ynu.edu.cn; 3 The International Joint Research Center for Sustainable Utilization of Cordyceps Bioresources in China and Southeast Asia, Yunnan University, Kunming 650091, China; 4 College of Pharmacy, Amar Shaheed Baba Ajit Singh Jujhar Singh Memorial College, Ropar 140111, Punjab, India; sandeep_pharm70@yahoo.com; 5 Department of Biosciences, Faculty of Sciences, COMSATS University Islamabad, Sahiwal 57000, Pakistan; saad.khan@cuisahiwal.edu.pk; 6 Department of Pharmacy and Pharmacology, School of Therapeutic Sciences, Faculty of Health Sciences, University of the Witwatersrand, Johannesburg 2193, South Africa

**Keywords:** hydrogel films, chitosan/PVA, honey, wound healing

## Abstract

In the present study, chitosan/polyvinyl alcohol (PVA)-based honey hydrogel films were developed for potential wound healing application. The hydrogel films were developed by a solvent-casting method and were evaluated in terms of thickness, weight variation, folding endurance, moisture content and moisture uptake. The water vapor transmission rate was found to range between 1650.50 ± 35.86 and 2698.65 ± 76.29 g/m^2^/day. The tensile strength and elongation at break were found to range between 4.74 ± 0.83 and 38.36 ± 5.39 N, and 30.58 ± 3.64 and 33.51 ± 2.47 mm, respectively, indicating significant mechanical properties of the films. SEM images indicated smooth surface morphology of the films. FTIR, DSC and in silico analysis were performed, which highlighted the docking energies of the protein–ligand complex and binding interactions such as hydrogen bonding, Pi–Pi bonding, and Pi–H bonding between the selected compounds and target proteins; hence, we concluded, with the three best molecules (lumichrome, galagin and chitosan), that there was wound healing potential. In vitro studies pointed toward a sustained release of honey from the films. The antimicrobial performance of the films was investigated against *Staphylococcus aureus*. Overall, the results signaled the potential application of chitosan/PVA based hydrogel films as wound dressings. Furthermore, in vivo experiments may be required to evaluate the clinical efficacy of honey-loaded chitosan/PVA hydrogel films in wound healing.

## 1. Introduction

Every year numerous patients suffer from different types of skin epidermal damage such as burns, ulcers, and other traumatic incidents leading to the development of acute and/or chronic wounds [1]. Wound healing is a complex phenomenon that includes the inflammation phase, proliferation phase and tissue remodeling phase. Traditional wound dressings such as cotton wool and gauze still possess the largest part of the wound dressing market. Polymeric wound dressings may include films, foams, hydrogels, hydrocolloids and fibers [2].

Hydrogels are three-dimensional polymeric networks that are capable of absorbing water without dissolving [3]. Hydrogels have been explored for significant applications in wound healing, drug delivery, water purification, tissue engineering, scaffoldings, and 3D printing [4]. The characteristic advantage of hydrogels is their high surface-area-to-volume ratio that allows rapid response and maximum interaction with the surrounding tissue [5].

Honey is a sweet, naturally derived material obtained from bees and some other insects. It has been used in the food industry and medicinal industry in past decades. It has strong antibiotic action against various microorganisms such as Gram-positive and -negative, and against methicillin-resistant *Staphylococcus aureus*. The activity of honey is mainly controlled by the content of a 1,2-dicarbonyl compound, known as methylglyoxal, where its concentration defines the manuka factor. Honey with a concentration above 0.15 mg/g of MGO has been considered to possess better antimicrobial and antioxidant properties. Momin et al. studied the use of honey and curcumin as sponges for wound healing applications [6]. El-kased et al. studied the use of honey-based hydrogels made of chitosan and carbopol 934 for burn wounds and antibacterial action against *Pseudomonas aeruginosa*, *Staphylococcus aureus*, *Streptococcus pyogenes* and *Klebsiella pneumonia* [7]. The manuka honey, when mixed with chitosan to form hydrogel, showed a dose-dependent effect; when the concentration of honey is varied, the swelling of the gel is increased [8].

Chitosan has been derived from marine sources that have been used on an exploitable scale in the design and formulation of dosage forms. It is semi-synthetic and derived from chitin. It has attracted researchers around the globe because of its biodegradable nature and can be molded into films, blends, coating, composites and nanotechnology-enabled profiles. Bagher et al. prepared a chitosan alginate-based hydrogel with 10% Hesperidin as a model drug for wound healing action [9]. Researchers also prepared chitosan-based antimicrobial wound healing hydrogels with mupirocin. The hydrogel was cross-linked with the monomer acrylamide-2-methyl propane sulfonic acid using *N*, *N*-methylene bisacrylamide as a cross linker [10]. Mndlovu et al. demonstrated the ability of chitosan to form interpolymer complexes with anionic polymers for developing wound dressing with tunable physical, chemical and mechanical properties [11].

Polyvinyl alcohol (PVA) is a vinyl polymer interconnected by carbon–carbon linkage. It is water-soluble and biodegradable. It also possesses high biocompatibility and is capable of self-crosslinking because of hydroxyl groups present on side chains. The PVA, along with cellulose linked with curcumin, has been used for wound healing activity [12]. Honey is a dark-colored liquid with strong antimicrobial properties. It has been reported to perform wound-healing action due to the presence of phenolic and flavonoid components [13]. Honey is a mixture of various chemicals available naturally, showing antimicrobial activity against both Gram-positive and Gram-negative properties. Apart from this, honey also performs anti-inflammatory activity [14]. Honey-based hydrogel using freeze-thawed technique were prepared using PVA/chitosan/Gelatin, which showed wound healing action [15]. Sangnim et al. developed a clindamycin-loaded nanofiber patch of PVA/tamarind gum, which exhibited the pronounced effect of the PVA concentration of properties, and performance of the formulation [16].

Hydrogels consist of a polymer matrix holding a large amount of aqueous media. Natural polymers, being not so competent at holding large amounts of water, require another second polymer to act as a helping polymer. Therefore, to compensate for the properties of Chitosan and sustain the release of the drug from hydrogel, PVA was used.

In the present study, honey-based hydrogel films, Chitosan/PVA-based, were formulated with the solvent-casting method. Various tests such as thickness, weight variation, folding endurance, moisture content, moisture uptake, swelling ratio, water vapor transmission rate, tensile strength, and elongation to break were performed for evaluating the quality of films for potential wound healing application. SEM, FTIR and DSC characterization studies were also performed. In vitro drug release, antimicrobial studies, stability testing and in silico testing were also carried out on the hydrogel film formulations. The novelty of research lies in the concept of developing physically crosslinked hydrogels of Chitosan and PVA without the use of any harmful organic chemical/solvent. 

## 2. Results and Discussion

### 2.1. Thickness, Weight Variation and Folding Endurance

**Figure 1:**
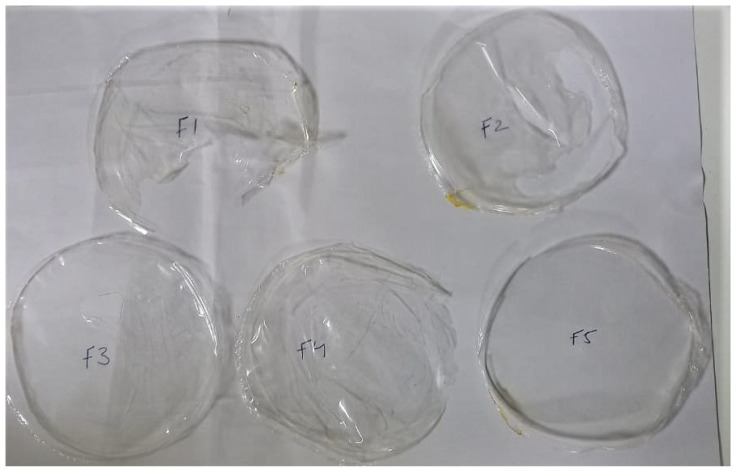
Pictorial Representation of various hydrogel films F1–F5.

The prepared hydrogel films were evaluated using various physicochemical parametric tests as depicted in Table 1. The evaluation tests are indicators of quality and reproducibility of the method for preparing the formulation. The thickness of the films was found to range between 0.041 ± 0.006 and 0.055 ± 0.004 mm. The weight variation was reported to range between 0.425 ± 0.02 and 0.480 ± 0.04 g. As the concentration of chitosan was increased from hydrogel film batch F1 to F5, the folding endurance was found to increase from 350 ± 15 to 445 ± 7. Films F1–F5 are shown in Figure 1.

### 2.2. Moisture Content and Moisture Uptake

The percentage of moisture content of the films was found to increase from 18.10 ± 1.05 to 24.22 ± 2.37% for batches F1 to F5. Cazón et al. reported that the presence of moisture improves the water vapor permeability, opacity and UV barrier properties of the films [17].

The moisture content of the hydrogel films was found to increase with an increasing concentration of chitosan. As chitosan has large amounts of the hydrophilic amino and hydroxyl groups, these could be held responsible for the absorption of excess water molecules. Similarly, moisture uptake by the hydrogel films was 11.35 ± 0.07 and 14.96 ± 0.06%. Moisture uptake is an important parameter for films used for wound healing application, as wound exudate soaking could be directly correlated with the moisture uptake property of the films.

### 2.3. Swelling Ratio

**Figure 2:**
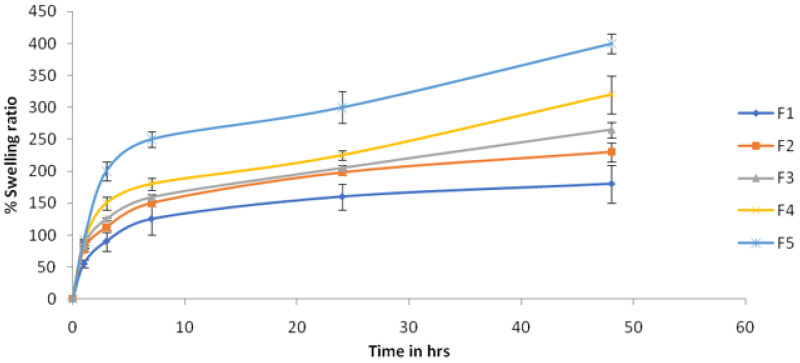
Swelling ratio hydrogel film batches (*n* = 3).

The swelling ratio was evaluated to study the fluid uptake capacity, which is an important parameter for elucidating the wound healing property of hydrogel films. The swelling ratio of water was found to increase as the concentration of chitosan was increased in the hydrogel films (Figure 2). An increase in the cross-linking density of the polymeric chains could be ascribed to increased chitosan content. Similar results were reported by Abdeen [18] and Casey [19] for the polymer-dependent swelling of hydrogels.

### 2.4. WVTR

WVTR is a parameter indicating the penetration of moisture through the film and is more important in food preservation to protect the material from moisture. The results of the WVTR analyses of different batches of hydrogel films are shown in Table 2. The WVTR of the films decreased significantly (*p* ≤ 0.05) when chitosan content was increased from batch F1 to F5. The WVTR was found to range between 1650.50 ± 35.86 and 2698.65 ± 76.29 g/m^2^/day. Kanatt et al. developed Chitosan/PVA-, based films for food packaging application and reported significant reduction in WVTR with an increasing content of chitosan [20]. Similar results were reported by Pelissari et al., in cassava starch–chitosan films, with the increasing concentration of chitosan WVTR found to decrease due to the formation of hydrogen bonds between the NH2 of Chitosan and OH of cassava starch, thereby reducing the availability of the hydrophilic groups [21]. Li et al. found that that an increase in chitosan concentration resulted in decreased WVTR of konjac glucomannane–chitosan films [22].

### 2.5. Tensile Strength and Elongation at Break

Tensile strength and elongation at break were selected as the parameters representing the mechanical properties of the hydrogel films. From the F1 to F5 batches of hydrogel films, tensile strength was found to range between 4.74 ± 0.83 and 38.36 ± 5.39 N. The elongation at break was found to range between 30.58 ± 3.64 and 33.51 ± 2.47 mm. Strong physical interactions and networking between chitosan and PVA could be responsible for the enhancement in mechanical properties of the hydrogel films. Good mechanical properties are a favorable feature for the industrial manufacturing, packaging, transportation and end-use application of hydrogel films. Chitosan membranes blended with PVA exhibited good mechanical properties for medical products and for controlled delivery of drugs [23].

### 2.6. FTIR

**Figure 3:**
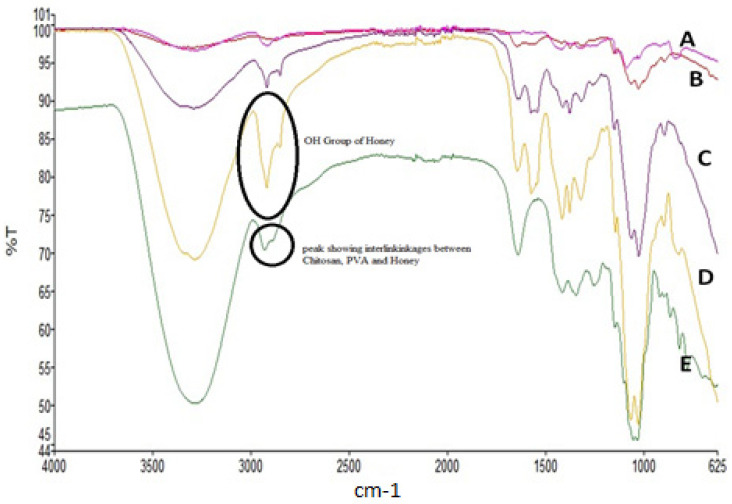
FTIR spectra of (**A**) PVA, (**B**) chitosan, (**C**) film with honey (formulation containing PVA/chitosan/honey), (**D**) honey, (**E**) film without honey (formulation containing PVA/chitosan).

The C-H alkyl stretching band was detected at 2922 cm^−1^ by FTIR, while the hydrogen-bonded band was detected at 3282 cm^−1^ for PVA. PVA hydrolysis was linked to the peak at 1711 cm^−1^, which was linked to the vibration of the -C=O group and the degree of hydrolysis of PVA. In the instance of Chitosan, the glucopyranose ring corresponded to the peak at roughly 900 cm^−1^. The bending vibration of the C-H group is reflected in the absorption peak at 1417 cm^−1^. C-N stretching vibrations accounted for the absorption peaks at 1658 cm^−1^ and 1320 cm^−1^, respectively. In the CH, the peak at 1028 cm^−1^ and 1060 cm^−1^ reflects the C-O stretching vibration. The signal at 1158 cm^−1^ is typical of glycosidic linkage based on the -C-O-C group. Stretch vibrations from carbohydrate, water and organic acids may be seen in honey’s FTIR spectrum at 3700 cm^−1^ and 3000 cm^−1^, respectively. C-H stretching vibrations are responsible for the 2929 cm^−1^ absorption band in sugar skeletons. The value of 1640 cm^−1^ was due to the existence of the bending vibrations of OH and stretching vibrations of the ketone functional group in Fructose and glucose, respectively. Carbohydrates have a chemical skeleton made up of C-O, C-C, and C-H, which causes stretching vibrations and bending of the C-H group to occur in the fingerprint region between 1450 and 700 cm^−1^. The films were found to have the same peak wave numbers for all FTIR spectra, however there was a little shift in peak wave numbers (Figure 3). The peak intensities fluctuated, although it is possible that this is due to linkage between Chitosan and PVA. A peak near 2850 cm^−1^ was seen in the FTIR of PVA after exposure to honey films, suggesting that honey played a part in the cross-linking process. A chitosan/PVA hydrogel film was studied by Abdeen 2011 using FTIR analysis to determine the molecular interactions responsible for its increased mechanical capabilities [18]. Chitosan/PVA films undergo reorganization of their aggregated structure due to strong electrostatic contact, as described by Liang et al. in an FTIR study [24].

### 2.7. SEM

**Figure 4:**
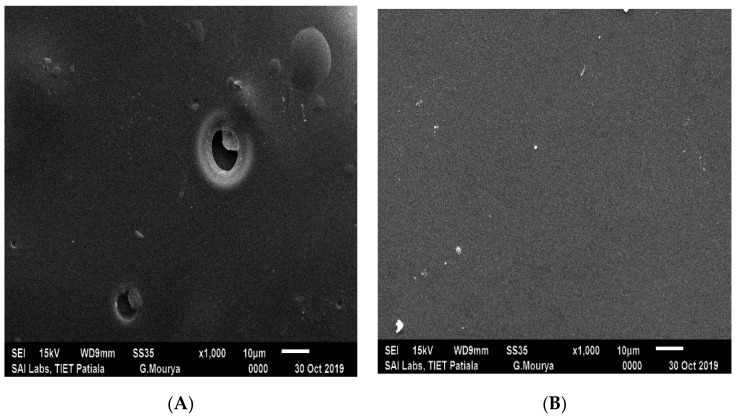
SEM images of hydrogel films: (**A**) F1 batch; (**B**) F5 batch.

SEM images indicated the surface morphology of the films as shown in Figure 4. The F5 formulation showed a relatively smooth surface, a homogenous matrix with fewer pores, as evidenced, or cracks exhibiting good structural integrity. Chitosan microdomains are evenly dispersed in the PVA matrix, forming a homogenous blend with good interfacial adhesion [25]. The formulation containing low amounts of Chitosan, i.e., F1, has a low binding density with the PVA polymer chain; as the concentration of Chitosan increased, i.e., F5, more chitosan was able to bind with PVA, leaving a smoother surface.

### 2.8. DSC

**Figure 5:**
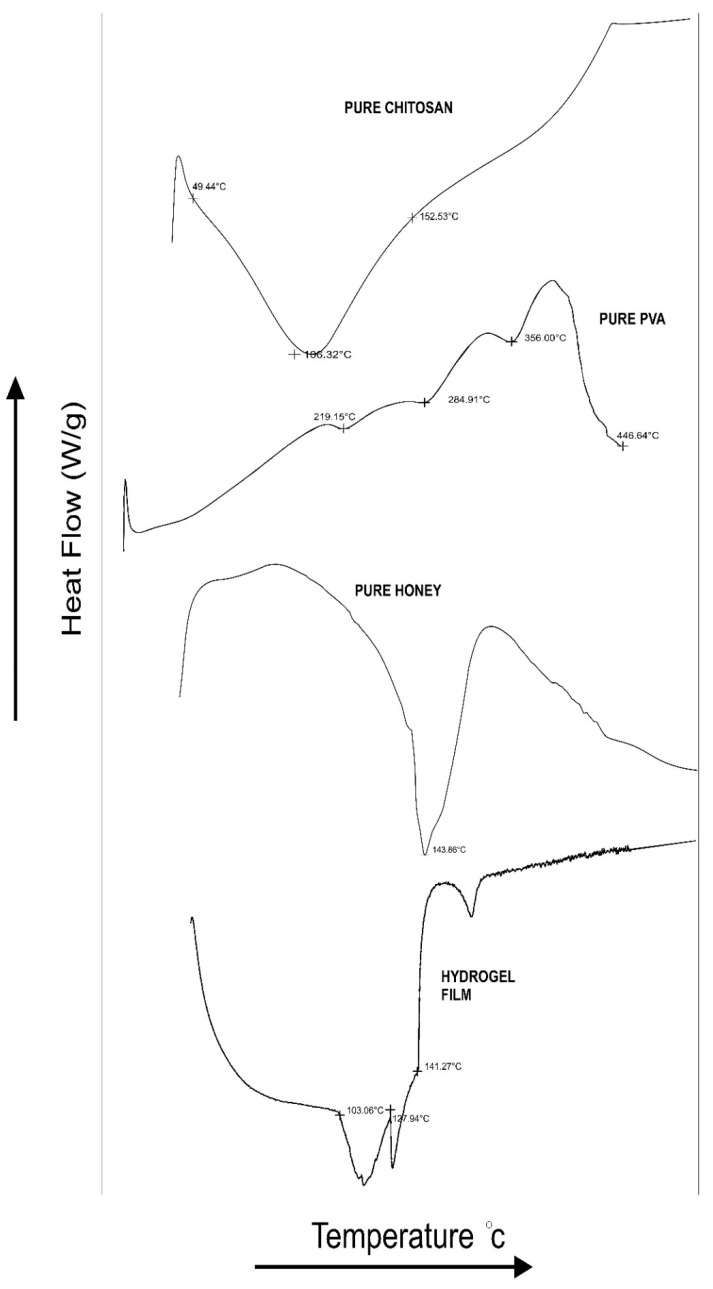
DSC thermograms of Chitosan, PVA, Honey, and Hydrogel film F5 batches.

The DSC thermograms of the Chitosan, PVA, honey and F5 hydrogel film batches are depicted in Figure 5. Characteristic endothermic peaks were exhibited by chitosan at 106.32 °C, PVA at 219.15 °C, and honey at 143.86 °C, indicating the melting point and purity of the respective compound. The appearance of endothermic peaks at 103.06 and 127.94 °C in the DSC thermogram of F5 hydrogel film batch indicates shifting of characteristic peaks due to subsequent bonding between chitosan and PVA, leading to the formation of a hydrogel matrix. The melting point of PVA was found to decrease with the increase in the concentration of chitosan, which could be ascribed to miscibility and subsequent bonding between the chitosan and PVA [26].

### 2.9. In Vitro Drug Release

**Figure 6:**
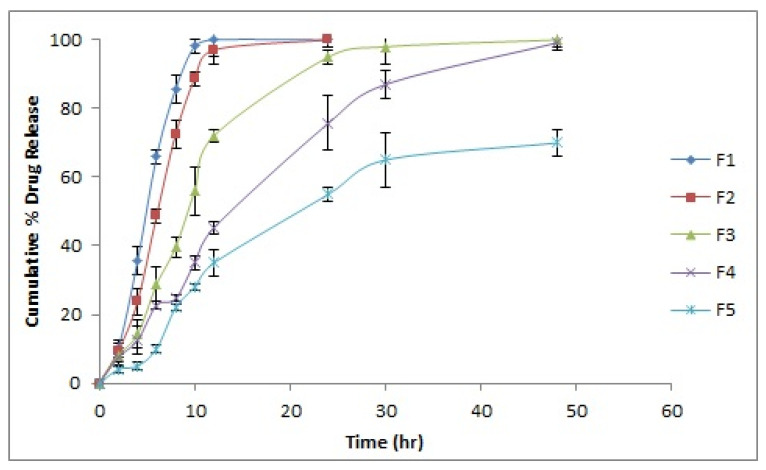
In vitro drug release profiles from different batches of hydrogel films.

The in vitro drug release from the different batches of formulated hydrogel films was indicative of a controlled release of honey for an extended period of time, which is significant for its pronounced wound healing effect (Figure 6). The effect of increasing the concentration of chitosan from F1 to F5 hydrogel films batches was pertinent in retarding the release of the therapeutic agent from within the films. The intermolecular networking between chitosan and PVA led to the formation of a strong matrix, which retards the release of the drug molecules. Similar results depicting the effect of increasing polymer concentration on drug release were reported by Kouchak et al. [27] and Wang et al. [23]

The in vitro drug release data were fitted to various releasing models, namely, the zero-order, first-order, Higuchi, Korsmeyer–Peppas, and Hixson–Crowell models (Table 3). Except for batch F1, the regression coefficients (r^2^) for all of the formulations suggests the Korsmeyer–Peppas model to be the best-fitted model. For formulations F1, F2 and F3, the value of n was found to range between 0.359 and 0.428, indicating Fickian diffusion to be the mechanism suggesting the release of the drug. In formulations F4 and F5 comprising relatively higher concentration of the polymer, the values of n were found to be 0.688 and 0.604, respectively, indicating the anomalous drug release mechanism that could be due to a complex phenomenon including the diffusion, erosion and relaxation of polymeric chains.

### 2.10. Antimicrobial Study

**Figure 7:**
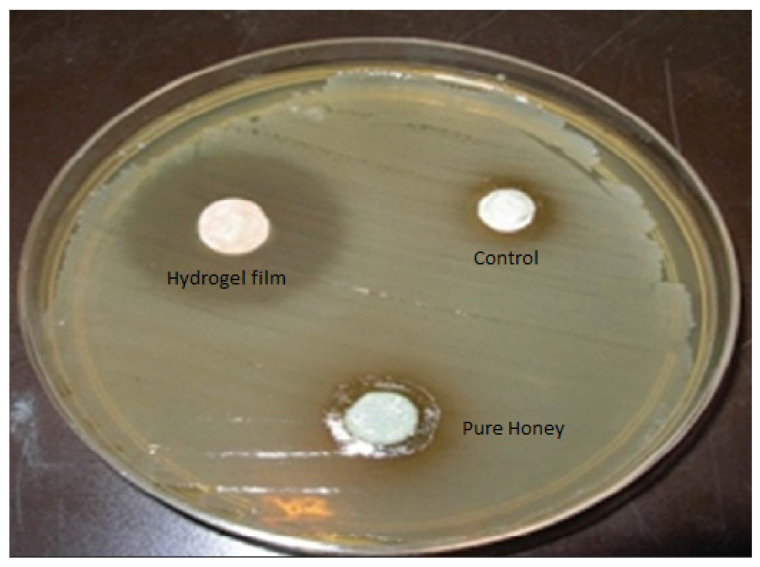
Microbial plates representing zone of inhibition for control, honey and hydrogel film.

The antibacterial performance of hydrogel films was investigated against *Staphylococcus aureus*. The zone of inhibition for formulation F4, control (untreated) and standard are depicted in Figure 7. The honey-based hydrogel film was found to exhibit significant antimicrobial efficacy with good bacteriostatic ability. Results are in line with the findings reported by other researchers [28,29]. The antibacterial action of the hydrogel dressing can be explained by the synergistic effect of chitosan and honey. When chitosan is dissolved in an acidic environment, the amino groups in the chains protonate into NH_3_^+^ and become cationic, allowing it to interact with various types of cell membranes. This positive charge is the main reason for the antimicrobial activity of chitosan. It interacts with the negatively charged cell membranes of the microorganisms, preventing their activity or resulting in cell death [30,31]. The antibacterial properties of honey depend on factors such as the osmotic effect, due to the high sugar content and low pH [32]. A low pH results from the presence of organic acids in honey [28]. The presence of honey in a chitosan hydrogel can result in synergistic antibacterial activity due to a lower pH and cationic charges. The diameter of the zone of inhibition for the F4 batch of hydrogel films against *Staphylococcus aureus* was 5.01 ± 0.32 mm, respectively. 

### 2.11. Stability Study

All of the formulated batches of the hydrogel films were subjected to stability testing as per the ICH guidelines. The test parameters, viz., folding endurance, moisture content, tensile strength and WVTR, were evaluated in the stability study (data shown in Table 4). No significant changes in the selected test parameters during the study period indicated good physical stability of the prepared hydrogel films.

### 2.12. Molecular Docking Investigations 

**Figure 8:**
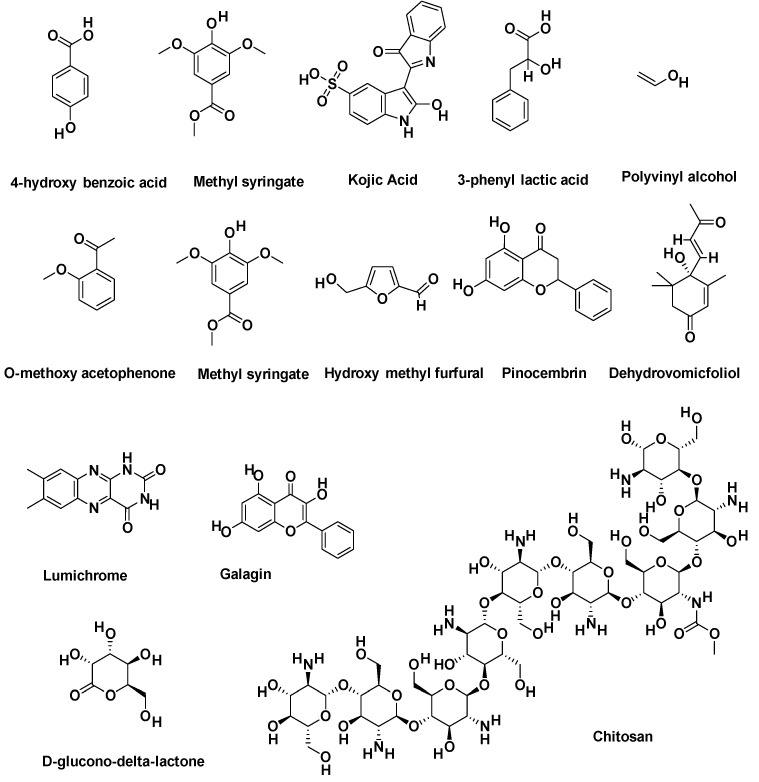
Dataset of 14 compounds used for molecular docking analysis.

**Figure 9:**
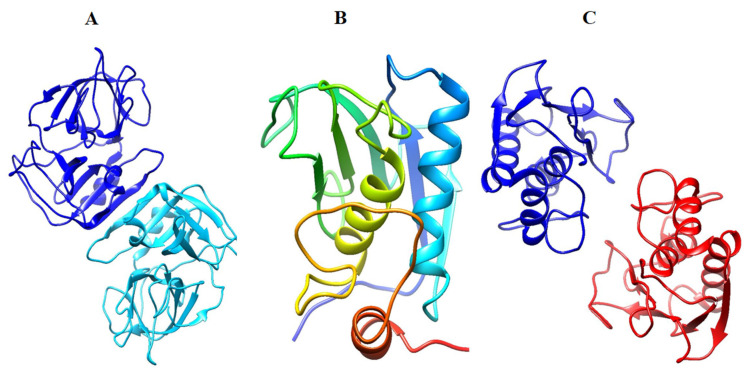
Three-dimensional representation of selected target proteins used for molecular docking: (**A**) human neutrophil elastase (HNE) (PDB ID: 1H1B); (**B**) matrix metalloproteinase-3 (MMP-3) (PDB ID: 1QIB); (**C**) matrix metallopeptidase 9 (MMP-9) (PDB ID: 4H1Q).

14 compounds were used for molecular docking, 12 of which were the honey extracts [33] and the other two of which were polyvinyl alcohol and chitosan; their two-dimensional chemical structures are highlighted in Figure 8. These compounds were subjected to MOE, which generated a .mdb extension-based database and performed protein–ligand docking simulations in the active site of the selected three proteins. Prepared protein conformations are shown in Figure 9. This prepared molecule database and proteins were used by the Dock module of MOE software for further docking investigations.

Table 5 lists the result summary of the 14 compounds docked in the vicinity of target proteins’ active binding sites in the range of 4.5 Å. Each compound presents a docked score in the form of Kcal/mol, as well as a calculation of the RMSD values for the best pose generated during molecular docking simulation by MOE. It is observed that for three target proteins—human neutrophil elastase (HNE) (PDB ID: 1H1B), matrix metalloproteinase-3 (MMP-3) (PDB ID: 1QIB) and matrix metallopeptidase 9 (MMP-9) (PDB ID: 4H1Q)—molecular docking results were in different ranges; some are moderate, but some are below the threshold value of the docking score, which is greater than or equal to −5.0. In addition, three compounds—Compound 11 (Lumichrome), 12 (Galagin) and 13 (Chitosan)—present the best results in terms of docking score and binding interactions.

Table 5 shows the 14 chemical compound names, Pubchem CID, docked score, and RMSD values, while Table 6 shows the summary of binding interactions of the top three docked complex results. Narayanaswamy et al., in their paper, highlighted the activity of 12 selected extracts of honey (compound 1–4, 6–12, 14) as potential HNE and MMP-2 and nine inhibitors [33], while we used these honey extracts to test the docking interactions with HNE and MMP-3 and nine protein targets, as mentioned in Table 6. Hence, the results revealed that honey extracts, lumichrome and galagin, presented the best-docked poses with good binding energies. Lumichrome is an industrially very important compound to assist in several pharmaceutical preparations, while Galagin is a flavonoid that has multiple bioactivities and a significant medicinal agent. The third compound; chitosan, is the most important wound healing agent, and is tested for its binding interaction with same three proteins. As suppression of these proteins promotes the wound healing mechanism, HNE, MMP-3 and MMP-9 inhibitors are beneficial for the recovery of acute and severe wounds [34,35]. Chitosan presents a very good docking score and binding interaction with the residues of the three target proteins, and acts as a significant inhibitor of the HNE, MMP-3 and MMP-9 target proteins.

**Figure 10:**
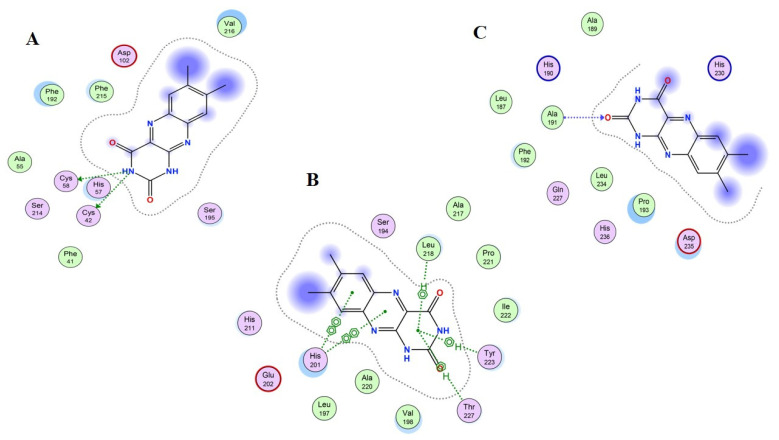
Schematic representation of Compound 11 “lumichrome” bonded in the active site of selected three proteins (PDB ID: 1H1B (**A**), 1QIB (**B**) and 4H1Q (**C**)). The active binding site shows hydrogen-bonding capacity as donor atoms (purple), and acceptor atoms (green), a two-dimensional plot showing hydrogen bonding interactions and other important hydrophobic interacting residues of the target proteins.

Figure 10 presents the best bond conformation of Compound 11, “lumichrome”, with the selected three proteins as (A)–(C). Figure 10A demonstrates that the N6 atom from compound 11 interacted with protein residues and generated two hydrogen bonds with the CYS42 and CYS58 residue of HNE protein, with a bond distance of 3.42 Å and a bond energy of −1.7 and −1.9 Kcal/mol; therefore, the docking score for the best binding pose was above the threshold value at −6.0911 Kcal/mol. Figure 10B demonstrates that the 6-ring atom of compound 11 generated six bonds with protein residues, four of which are Pi–hydrogen bonds and two of which are Pi–Pi bonds, and the ligand bonded with MMP-3 protein presented very good docking score of −7.3801 Kcal/mol. Figure 10C demonstrates that compound 11 generated one hydrogen bond, and the O2 ligand atom interacted and generated a hydrogen bond with a nitrogen atom of the ALA191 residue of the MMP-9 protein, with a processes docking score of −6.9001 Kcal/mol.

**Figure 11:**
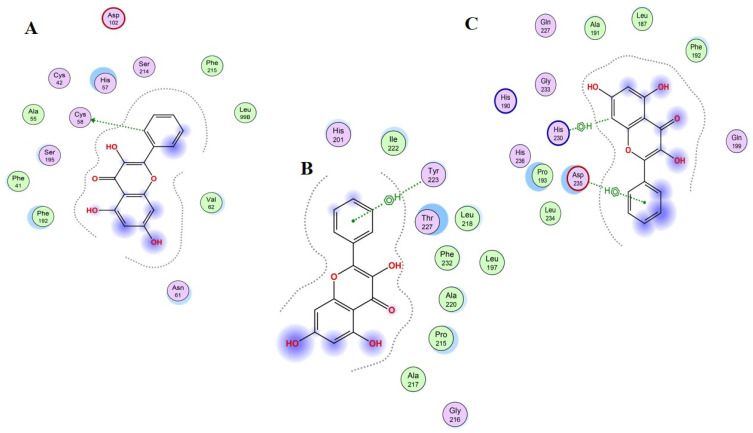
Schematic representation of Compound 12 “galagin” bonded in the active site of selected three proteins (PDB ID: 1H1B (**A**), 1QIB (**B**) and 4H1Q (**C**)). The active binding site shows hydrogen-bonding capacity as donor atoms (purple), and acceptor atoms (green), a two-dimensional plot showing hydrogen bonding interactions and other important hydrophobic interacting residues of the target proteins.

Figure 11 presents the best bond conformation of Compound 12, “galagin”, with the selected three proteins as (A)–(C). Figure 11A demonstrates that the C16 atom of compound 12 interacted with residues of the active site of the target protein, and generated one hydrogen bond with the CYS58 residue of the HNE protein, within a distance range of 3.82 Å, and a bond energy of −0.5 Kcal/mol. Therefore, the best bond conformation of the protein–ligand complex was generated with energy (dock score = −6.0133 Kcal/mol). Figure 11B demonstrates that the 6-ring atom of compound 12, involved in binding interactions, generated one Pi-bond with a carbon atom of the TYR223 residue of the MMP-3 protein. The bond distance range was 4.72 Å and the best binding conformation was generated with energy (dock score = −7.3211 Kcal/mol). C13 and the 6-ring atom of compound 12 was involved in binding interactions and generated a Pi-bond with the 5-ring of the His230 residue, and another Pi-bond with carbon of the ASP235 residue of the MMP-9 protein; the estimated docking score is −6.8556 Kcal/mol (Figure 11C).

**Figure 12:**
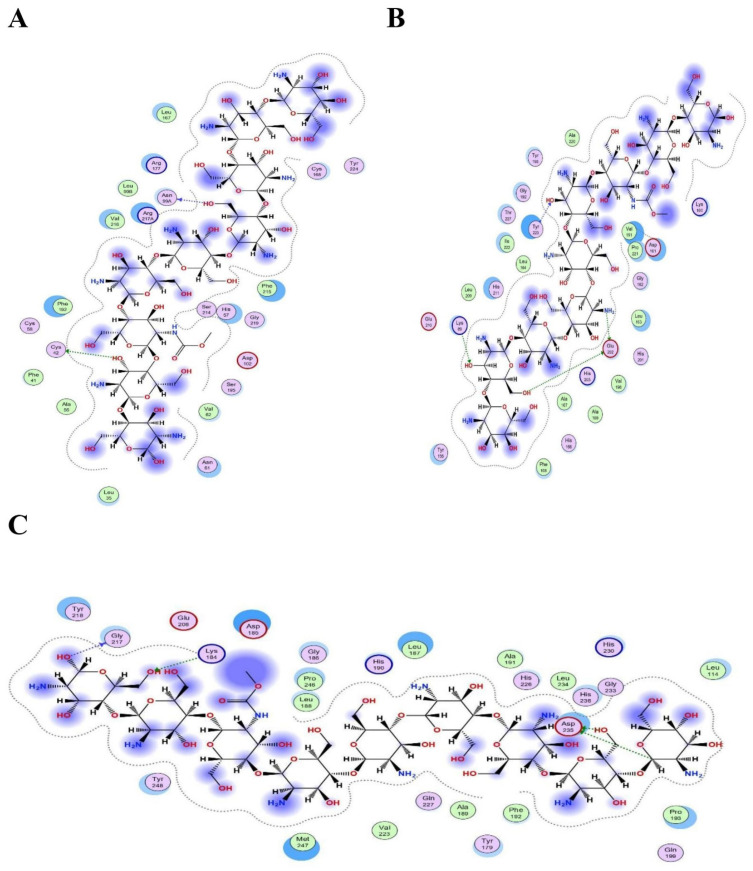
Schematic representation of Compound 13 “chitosan” bonded in the active site of selected three proteins (PDB ID: 1H1B (**A**), 1QIB (**B**) and 4H1Q (**C**)). The active binding site shows hydrogen bonding capacity as donor atoms (purple), and acceptor atoms (green), a two-dimensional plot showing hydrogen bonding interactions and other important hydrophobic interacting residues of the target proteins.

Figure 12 presents the best bond conformation of compound 13, “chitosan”, with the selected three proteins as (A)–(C). Figure 12A demonstrates that compound 13 was involved in an interaction with an O21 and an O28 atom, and generated two hydrogen bonds with the CYS42 and ASN99 residues of the HNE protein; it processed a docking score above the threshold value, the best of all docked results at −11.8369 Kcal/mol. Figure 12B demonstrates that compound 13 was involved in a binding interaction with O32, O43, O15, and O22 atoms, and generated four hydrogen bonds with the GLU202, TYR223, and LYS89 residues of the MMP-3 protein, with a very good docking score of −11.6352 Kcal/mol. Figure 12C demonstrates that compound 13 was involved in a binding interaction with O32, O35, C90, and O37 atoms, and generated four hydrogen bonds with the ASP235, GLY217, ASP235, and LYS184 residues of the MMP-9 protein. The processed docking score was −12.8897 Kcal/mol [36].

## 3. Conclusions

Chitosan/PVA-based hydrogel films loaded with honey were successfully developed for potential wound healing application. The films exhibited significant swelling, moisture uptake and mechanical properties which are ideally required for a good wound dressing formulation. SEM, FTIR and DSC studies were performed for studying the surface morphology and molecular interactions of the polymer used to formulate films. In vitro release of honey from the hydrogel films indicated its role for its use for developing a controllable drug delivery system for wound healing application. The in silico studies showed the interaction of honey and its constituent components with the proteins involved in wound healing. Additionally, an ADMET profile was estimated that explains the structure-to-activity guide of the three best polymers, which could be helpful in their synthesis and in clinical experiments. Furthermore, in vivo experiments could be significant in evaluating the clinical efficacy of honey-loaded Chitosan/PVA hydrogel films in wound healing.

## 4. Material and Methods

### 4.1. Materials

Chitosan (Low molecular weight, 50,000–190,000 Da, 75.0% Deacetylated) was purchased from Sigma Aldrich, USA. PVA (molecular weight, approx. 115,000 Da, 98.9 mole percent hydrolyzed) was purchased from Loba Chemicals Pvt. Ltd. Mumbai, India. Honey was procured from the local market of Patiala, Punjab, India. All other reagents and chemicals were of analytical grade.

### 4.2. Preparation of Hydrogel Films

The hydrogel films were prepared by a solvent-casting method. Chitosan solutions (different concentrations as shown in Table 7) were prepared by dissolving chitosan in acetic acid solution (3% *v*/*v*) with constant stirring for 2 h. PVA solution (5% *w*/*v*) was prepared by dissolving PVA in distilled water with constant stirring at 50 °C for 4 h. The chitosan and PVA solutions were combined with honey in variable proportions, as given in Table 1, with mechanical blending at 1000 rpm for 10 min. The mixture (0.5 g) was transferred into Pyrex petri plates (5 inch diameter) and allowed to air dry at normal room conditions for 2 days. Dried films were then peeled from the petri plates and stored in a desiccator for further use.

### 4.3. Thickness and Weight Variation

The thickness of hydrogel films was recorded using a digital calibrated micrometer (Mitutoyo, Japan). The average and standard deviation of the three readings were recorded. For the weight variation test, the films were weighed individually, and results were determined using the average ± SD. The evaluations were performed in triplicate [37].

### 4.4. Folding Endurance 

The folding endurance was evaluated to verify the number of times the film can be folded. The number of times a film sample could be folded at the same place without breaking indicated the folding endurance value. The experiment was performed in triplicate [37].

### 4.5. Moisture Content

The hydrogel films were initially weighed (Wi) and were placed in a desiccator containing activated silica gel for 24 h. The films were weighed repeatedly until a constant weight (Wd) was observed. The moisture content was determined as per the following equation:

Moisture Content (%) = (Wi − Wd)/Wd × 100

The moisture content determination experiment was performed in triplicate [38].

### 4.6. Moisture Uptake

The hydrogel films were initially weighed (Wi) and were placed in a desiccator containing activated silica gel for 24 h. The films were transferred to another desiccator for 72 h containing saturated sodium chloride solution with relative humidity maintained at 75%.The final weight of the films (Wm) was recorded and the moisture uptake capacity was determined according to the equation given below:

Moisture uptake (%) = (Wm − Wi)/Wi × 100

The moisture uptake experiment was performed in triplicate.

### 4.7. Swelling Ratio

The dried hydrogel films were cut into square-shaped specimens (2 cm × 2 cm). The samples were weighed and immersed in 250 mL of phosphate buffer (pH 7.4) at 25 °C. At predetermined time intervals, the film samples were weighed after blotting with tissue paper to remove the surface water. The swelling ratio was calculated by the following formula:

Swelling ratio (%) = (W_s_ − W_d_)/W_d_ × 100

where, W_d_ is the initial weight of the dry film samples and W_s_ is the weight of swollen film samples. The experiment was performed in triplicate.

### 4.8. Water Vapor Transmission Rate (WVTR)

The WVTR test was performed as per ASTM D6701-21 [38]. The sample film was mounted on the top of a polytop glass (144 mm^2^) containing a phosphate buffer of 10 mL (pH 7.4). The sample films were pre-weighed and put in an oven for 24 h at 35 °C. Using the following equation, WVTR was determined.

WVTR = W_i_ − W_t_/A × 10^6^ g/m^2^day^−1^

where, A = polytop opening area (mm^2^), Wi and Wt = polytop weight before and after being put in the oven, respectively. The experiment was performed in triplicate.

### 4.9. Mechanical Properties

The samples of hydrogel films were analyzed for mechanical properties (tensile strength (N/mm^2^) and elongation at break (%)) by texture analyzer (TA XT plus, Stable Microsystem, Godalming, UK) with 5 kg of loaded cell. Film of size 1 cm^2^ was cut and clutched between the clamps followed by separation at rate of 50 mm/min. The experiment was performed in triplicate [37].

### 4.10. Fourier-Transform Infrared Spectroscopy (FTIR) 

The possible interactions between different components of films were evaluated by FTIR. The Infrared spectroscopy was performed on vacuum-desiccated hydrogel film and chitosan, PVA, and honey using Perkin–Elmer (Spectrum two, Model no.L160000A, Waltham, MA, USA), and scanning between 4000–650 cm^−1^ at a resolution of 4 cm^−1^ [37].

### 4.11. SEM

Morphological examination of the hydrogel films was performed by scanning electron microscope (S 4300 SE/N, Hitachi, Tokyo, Japan), with an accelerating voltage of 15 kV. All of the samples were staged on a metallic stub adhered with double side tape and further coated with a golden layer.

### 4.12. DSC

For DSC (Mettler Toledo star 1, Switzerland) analysis, the samples were placed and sealed in aluminum pans followed by heating at ambient temperature from 50 to 300 °C at a pre-programmed heating rate of 10 °C min^−1^.

### 4.13. In Vitro Drug Release 

The sample hydrogel films of fixed dimensions were mounted to a glass slide and affixed to a mesh screen of stainless steel. This assembly was securely placed at the bottom of the dissolution test apparatus (Paddle type- Lab India DS 8000, New Delhi, India), using a phosphate buffer of pH 7.4 as dissolution media, at a temperature of 37  ±  0.5 °C and a 50 rpm paddle speed. Aliquot samples (5 mL) were withdrawn at specified time intervals and the concentration of honey released was analyzed by UV spectrophotometric analysis at 500 nm employing a calibrated UV/Visible spectrophotometer (2202, Systronics, India). In vitro drug release data were fitted into various kinetic models, viz., zero-order, first-order, Higuchi, Hixson–Crowell and Korsmeyer–Peppas model, for understanding the mechanism of drug release from the formulation.

Zero-order: $Q=Q_{o}+k_{o}t$

First-order: $\ln Q=\ln Q_{o}+k_{1}t$

Higuchi model: Q=kHt12

Hixson-Crowell model: Qo13−QR13=Kst

Korsmeyer-Peppas model: QQt=Kkptn

Where *Q* is the amount of drug release at time t, *Q_0_* is the initial amount of drug, *Q_R_* is the amount of drug remaining at time *t*, and *Q_t_* is the total amount of drug release. *k_0_*, k_1_, *k_H_* and *k_KP_* are the kinetic constants for the zero-order, first-order, Higuchi, Hixson–Crowell and Korsmeyer–Peppas models, respectively, and n is the release exponent. The in vitro dissolution experiment was performed in triplicate.

### 4.14. Antimicrobial Study

In vitro antibacterial performance of hydrogel films was evaluated by the disc diffusion method against the *Staphylococcus aureus* microorganism. The freshly grown inoculum of bacteria (10^6^ cells, prepared using serial dilution method) was seeded with 100 µL of freshly prepared tryptic soy agar media and the plates were incubated for 2 days at 32 °C. The plates were removed from the incubator and yellow-colored colonies were formed for *Staphylococcus aureus.* The hydrogel film was applied on the bacterial colonies, while one colony was taken as control (without disc) and incubated at 37 °C for 24 h, followed by the measurement of the diameter of the inhibition zone.

### 4.15. Stability Study

All of the formulated batches of the hydrogel films were subjected to stability analysis in accordance with ICH guidelines by storing them at a temperature of 40 ± 2 °C and in 75 ± 5% relative humidity conditions for a period of 3 months. The hydrogel film samples were wrapped in aluminum foil and placed in the stability chamber (Remi, India) under the mentioned conditions. The samples were withdrawn after 1, 2 and 3 months and were evaluated for physicochemical properties such as folding endurance, moisture content, tensile strength and WVTR [38].

### 4.16. Construction of Chemical Database for In Silico Screening 

A database of 14 chemical compounds was collected from a literature search, including Chitosan, Polyvinyl alcohol, and 12 metabolites of honey. Their chemical structures were retrieved from the PubChem Database for further investigations [39].

### 4.17. Protein Target Selection

Three proteins involved in wound healing—human neutrophil elastase (HNE) (PDB ID: 1H1B) [40], matrix metalloproteinase-3 (MMP-3) (PDB ID: 1QIB) [41] and matrix metallopeptidase 9 (MMP-9) (PDB ID: 4H1Q) [42]—were selected based on their resolution, and a ligand attached at their respective catalytic sites to perform a molecular docking experiment in this study, to evaluate the protein–ligand binding interactions.

### 4.18. Protein–Ligand Docking and Interactions Analysis

Molecular docking is an important computer-aided drug design and discovery application for the identification of protein–ligand interactions to understand the molecular mechanism of small drug-like entities in cellular pathways [43]. Fourteen compounds were used for molecular docking, and the three-dimensional structure of the selected proteins (PDB ID: 1H1B, 1QIB and 4H1Q) in .pdb format was imported to the molecular operating environment (MOE) software [44]. Heteroatoms, 3D protonation, and water molecules, along with the default ligand attached to the target protein, were removed to prepare proteins for the docking procedure. An active site in each protein structure was identified on the basis of previous literature [40,41,42], and structural optimization was performed by following the parameters; like the addition of hydrogen atoms, energy minimization with the Amber14 force field method was applied with chiral constraints and geometrical parameters. By using the Surfaces and Maps panel module, the transparency of the front and the back surface was adjusted and resulted in the information of key residues in the selected substrate binding site of each protein, in the native conformation. MOE software created a database of 14 compounds identified from experimental studies to perform molecular docking simulations, and saved them with a .mdb extension for further analysis. The top-ranked poses were subjected for refinement and calculation of binding free energies (ΔG), which was evaluated by scoring function (GBVI/WSA dg) [45]. A reliable scoring scheme that results in the docking score of the correct binding poses was established by the number of molecular interactions (hydrogen, Pi, and hydrophobic interactions) [46]. The MOE database of the docked complex was visualized carefully to understand the mode of binding interactions of α-glucosidase inhibitors bound in the selected pocket of the target protein.

### 4.19. Statistical Analysis

All experiments were carried out in triplicate and the average values with standard errors were reported. The data of various measured values were collected, tabulated and analyzed by using one-way ANOVA and level of significance (5%), *p* value (*p* ≤ 0.05), using SPSS software version 27.0.1.

## Figures and Tables

**Table 1:** Results of Physicochemical evaluation of hydrogel films. (*n* = 3).

Formulation Code	Thickness (mm)	Weight Variation (g)	Folding Endurance	Moisture Content (%)	Moisture Uptake (%)
F1	0.052 ± 0.003	0.462 ± 0.09	350 ± 15	18.10 ± 1.05	11.35 ± 0.07
F2	0.046 ± 0.006	0.429 ± 0.06	405 ± 9	12.52 ± 1.14	11.95 ± 1.01
F3	0.055 ± 0.004	0.480 ± 0.04	430 ± 11	17.38 ± 2.56	12.25 ± 0.08
F4	0.041 ± 0.006	0.425 ± 0.02	433 ± 10	21.57 ± 1.93	13.65 ± 0.09
F5	0.048 ± 0.007	0.447 ± 0.08	445 ± 7	24.22 ± 2.37	14.96 ± 0.06

**Table 2:** Tensile strength, elongation at break and WVTR parameters for batches of hydrogel films (*n* = 3).

Formulation Code	Tensile Strength (N)	Elongation at Break (mm)	WVTR (g/m^2^/day)
F1	4.74 ± 0.83	30.58 ± 3.64	2698.65 ± 76.29
F2	10.52 ± 1.45	31.10 ± 4.56	2458.87 ± 71.40
F3	23.77 ± 3.85	31.62 ± 5.25	2150.66 ± 80.19
F4	25.15 ± 2.66	31.98 ± 3.09	1911.53 ± 55.41
F5	38.36 ± 5.39	33.51 ± 2.47	1650.50 ± 35.86

**Table 3:** In vitro dissolution data fitted to various models.

Formulation Code	Zero-Order		First-Order		Higuchi		Korsmeyer–Peppas			Hixson–Crowell	
	r^2^	k_0_	r^2^	k_1_	r^2^	k_H_	r^2^	k_KP_	n	r^2^	k_HC_
F1	0.981	10.67	0.842	−0.161	0.978	3.318	0.975	0.637	0.428	0.94	−0.342
F2	0.981	8.898	0.873	−0.122	0.98	3.032	0.987	0.582	0.359	0.945	−0.273
F3	0.926	4.441	0.912	−0.087	0.966	2.201	0.961	0.581	0.403	0.973	−0.16
F4	0.923	2.212	0.945	−0.041	0.961	1.507	0.976	0.638	0.688	0.997	−0.078
F5	0.891	2.087	0.993	−0.028	0.934	1.475	0.945	0.659	0.604	0.98	−0.064

**Table 4:** Stability testing data for the prepared hydrogel films.

Batch	Time Interval (Months)	Test Parameters			
		Folding Endurance	Moisture Content (%)	Tensile Strength (N/mm^2^)	WVTR (g/m^2^/day)
F1	0	350 ± 15	18.10 ± 1.05	4.74 ± 0.83	2698.65 ± 76.29
	1	348 ± 14	18.10 ± 1.01	4.70 ± 0.59	2695.54 ± 77.58
	2	349 ± 17	18.10 ± 1.09	4.71 ± 0.78	2696.60 ± 75.41
	3	350 ± 17	18.10 ± 1.12	4.74 ± 0.65	2697.54 ± 74.57
F2	0	405 ± 9	12.52 ± 1.14	10.52 ± 1.45	2458.87 ± 71.40
	1	403 ± 6	12.45 ± 1.18	10.51 ± 1.49	2459.65 ± 70.45
	2	404 ± 7	12.50 ± 1.11	10.48 ± 1.50	2454.98 ± 71.50
	3	401 ± 8	12.54 ± 1.16	10.49 ± 1.40	2452.45 ± 70.45
F3	0	430 ± 11	17.38 ± 2.56	23.77 ± 3.85	2150.66 ± 80.19
	1	429 ± 10	17.36 ± 2.41	23.72 ± 3.80	2149.65 ± 79.74
	2	428 ± 9	17.32 ± 2.52	23.74 ± 3.79	2151.46 ± 79.85
	3	430 ± 10	17.38 ± 2.50	23.71 ± 3.75	2148.85 ± 79.90
F4	0	433 ± 10	21.57 ± 1.93	25.15 ± 2.66	1911.53 ± 55.41
	1	432 ± 9	21.51 ± 1.93	25.13 ± 2.61	1912.33 ± 55.41
	2	430 ± 9	21.48 ± 1.90	25.15 ± 2.60	1911.65 ± 55.41
	3	431 ± 8	21.49 ± 1.89	25.14 ± 2.65	1913.54 ± 55.41
F5	0	445 ± 7	24.22 ± 2.37	38.36 ± 5.39	1650.50 ± 35.86
	1	444 ± 7	24.20 ± 2.35	38.34 ± 5.37	1648.49 ± 34.54
	2	444 ± 6	24.15 ± 2.31	38.31 ± 5.30	1649.65 ± 33.12
	3	443 ± 7	24.18 ± 2.38	38.34 ± 5.35	1651.45 ± 35.65

**Table 5:** Summary of molecular docking results performed by molecular operating environment (MOE).

Sr. No	Compound Names	PubChem CID	Protein PDB ID: 1H1B		Protein PBD ID: 1QIB		Protein PBD ID: 4H1Q	
			Score	RMSD	Score	RMSD	Score	RMSD
1	4-hydroxybenzoic acid	135	−4.1647	1.5935	−4.9175	1.5940	−4.9309	1.4805
2	Methyl syringate	880	−3.6409	2.1469	−3.7874	1.1233	−3.8436	1.1154
3	Kojic Acid	3708	−5.7348	1.1504	−7.1493	1.6731	−7.3917	0.6537
4	3-phenyl lactic acid	3848	−4.6707	0.8809	−5.5804	1.4010	−5.7754	1.6327
5	Polyvinyl alcohol	11199	−3.1533	3.0526	−3.1736	0.9737	−3.1732	3.4360
6	O-methoxyacetophenone	68481	−4.7006	0.9142	−5.1265	0.8240	−5.4133	1.1089
7	Methyl syringate	70164	−5.1639	1.1198	−5.7789	1.5015	−6.3107	0.7194
8	Hydroxymethylfurfural	237332	−4.382	1.6009	−4.7615	1.2097	−4.8671	2.7057
9	Pinocembrin	238782	−5.0966	2.2995	−6.3780	1.1495	−6.7934	1.7762
10	Dehydrovomifoliol	688492	−5.9192	2.5804	−6.0208	0.7519	−5.7665	2.1204
11	Lumichrome	5326566	−6.0911	1.1315	−7.3801	1.0649	−6.9001	0.8905
12	Galagin	5281616	−6.0133	0.8527	−7.3211	1.3412	−6.8556	1.8762
13	Chitosan	71853	−11.8369	4.7772	−11.6352	4.0294	−12.8897	3.7408
14	D-glucono delta-lactone	7043900	−4.7835	0.6276	−5.2760	0.9685	−5.4439	0.9102

**Table 6:** Summary of binding interaction of the best hits with selected proteins from the database of 14 compounds.

Compound	Dock Score	Interacting Residues in the Binding Pocket				
		Ligand	Receptor	Interaction	Distance	E (kcal/mol)
Human Neutrophil Elastase (HNE) (PDB ID: 1H1B)						
Lumichrome	−6.0911	O6 O6	SG:CYS42(A) SG:CYS58(A)	H-donor H-donor	3.42 3.42	−1.7 −1.9
Galagin	−6.0133	C16	SG:CYS58(A)	H-donor	3.82	−0.5
Chitosan	−11.8369	O21 O28	SG:CYS42(A) O:ASN99(A)	H-donor H-donor	3.93 2.89	−0.5 −1.1
Matrix metalloproteinase-3 (MMP-3) (PDB ID: 1QIB)						
Lumichrome	−7.3801	6-ring 6-ring 6-ring 6-ring 6-ring 6-ring	CD1:LEU218(A) CA:TYR223(A) CD1:TYR223(A) N:THR227(A) 5-ring:HIS201(A) 5-ring:HIS201(A)	Pi–H Pi–H Pi–H Pi–H Pi–Pi Pi–Pi	4.45 4.03 4.30 4.22 3.75 3.57	−0.6 −1.2 −0.6 −3.9 −0.0 −0.0
Galagin	−7.3211	BR2 5-ring	OE2:Glu202(A) CA:LEU218(A)	H-donor Pi–H	3.45 3.93	−0.9 −1.6
Chitosan	−11.6352	O32 O43 O15 O22	OE1:GLU202(A) OE2:GLU202(A) N:TYR223(A) NZ:LYS89(A)	H-donor H-donor H-acceptor H-acceptor	2.79 2.84 2.87 316	−3.7 −1.7 −1.9 −2.4
Matrix metallopeptidase 9 (MMP-9) (PDB ID: 4H1Q)						
Lumichrome	−6.9001	O2	N: ALA191(A)	H-acceptor	3.38	−0.5
Galagin	−6.8556	C13 6-ring	5-ring: HIS230(A) CB:ASP235(A)	H–Pi Pi–H	3.61 3.75	−1.2 −1.1
Chitosan	−12.8897	O32 O35 C90 O37	OD2:ASP235(A) O:GLY217(A) OD2:ASP235(A) NZ:LYS184(A)	H-donor H-donor H-donor H-acceptor	3.04 3.08 3.05 3.43	−2.3 −0.6 −1.4 −1.4

**Table 7:** Composition table for preparing different batches of hydrogel films.

Formulation Code	Chitosan Solution		PVA Solution (5% *w*/*v*) (mL)	Honey (g)
	Concentration of Chitosan (% *w*/*v*)	Amount of Chitosan (mL)		
F1	0.25	80	20	1
F2	0.50	80	20	1
F3	0.75	80	20	1
F4	1.0	80	20	1
F5	2.0	80	20	1

## Data Availability

Not applicable.
